# E-cigarette Product Use, or Vaping, Among Persons with Associated Lung Injury — Illinois and Wisconsin, April–September 2019

**DOI:** 10.15585/mmwr.mm6839e2

**Published:** 2019-10-04

**Authors:** Isaac Ghinai, Ian W. Pray, Livia Navon, Kevin O’Laughlin, Lori Saathoff-Huber, Brooke Hoots, Anne Kimball, Mark W. Tenforde, Jennifer R. Chevinsky, Mark Layer, Ngozi Ezike, Jonathan Meiman, Jennifer E. Layden

**Affiliations:** ^1^Illinois Department of Public Health; ^2^Epidemic Intelligence Service, CDC; ^3^Wisconsin Department of Health Services; ^4^Division of State and Local Readiness, Center for Preparedness and Response, CDC; ^5^Division of Unintentional Injury Prevention, National Center for Injury Prevention and Control, CDC; ^6^National Center for Environmental Health, CDC; ^7^Emory University School of Medicine, Atlanta, Georgia.

In July 2019, the Illinois Department of Public Health and the Wisconsin Department of Health Services launched a coordinated epidemiologic investigation after receiving reports of several cases of lung injury in previously healthy persons who reported electronic cigarette (e-cigarette) use, or vaping (*1*). This report describes features of e-cigarette product use by patients in Illinois and Wisconsin. Detailed patient interviews were conducted by telephone, in person, or via the Internet with 86 (68%) of 127 patients. Overall, 75 (87%) of 86 interviewed patients reported using e-cigarette products containing tetrahydrocannabinol (THC), and 61 (71%) reported using nicotine-containing products. Numerous products and brand names were identified by patients. Nearly all (96%) THC-containing products reported were packaged, prefilled cartridges, and 89% were primarily acquired from informal sources (e.g., friends, family members, illicit dealers, or off the street). In contrast, 77% of nicotine-containing products were sold as prefilled cartridges, and 83% were obtained from commercial vendors. The precise source of this outbreak is currently unknown (*2*); however, the predominant use of prefilled THC-containing cartridges among patients with lung injury associated with e-cigarette use suggests that they play an important role. While this investigation is ongoing, CDC recommends that persons consider refraining from using e-cigarette, or vaping, products, particularly those containing THC. Given the diversity of products reported and frequency of patients using both THC- and nicotine-containing e-cigarette products, additional methods such as product testing and traceback could help identify the specific cause of this outbreak.

During July–September 2019, possible cases of lung injury associated with e-cigarette use in Illinois and Wisconsin were investigated to determine symptoms, exposures, and medical care history related to the outbreak. Patients were classified as having confirmed or probable cases of lung injury associated with e-cigarette use according to CDC’s interim outbreak case definitions (*3*). Interviews were conducted with patients or a proxy using a structured and scripted questionnaire that was developed jointly between Illinois and Wisconsin with guidance from CDC. The questionnaire asked detailed questions about e-cigarette use, including the names of e-cigarette, or vaping, products and devices, frequency of use, and product sources in the 3 months preceding illness onset. Most interviews were conducted by state or local health department staff members or in person by health care facility staff members during a patient’s hospitalization; a small number of patients completed the same survey online. In total, 86 (68%) interviews were completed among the 127 confirmed and probable patients that had been identified in Illinois (75) and Wisconsin (52) as of September 20, 2019.

Among the 86 confirmed and probable patients that were interviewed, including 48 from Illinois and 38 from Wisconsin, 68 (79%) were male, and the median age was 21 years (range = 15–53 years) (Table 1). Hospitalization dates among patients were similar in Illinois and Wisconsin, ranging from April 24 to September 19, 2019, and closely reflected the national outbreak (*2*). Illinois cases predominantly occurred in the northeast region of the state (in Chicago and the surrounding counties, close to the Wisconsin border) but have since been reported in other regions of the state. Most Wisconsin cases were initially clustered in the southeastern region of the state but have since been reported throughout western and central Wisconsin as well. 

**TABLE 1:** Patient characteristics by type of electronic cigarette, or vaping, product used in the 3 months prior to illness onset — Illinois and Wisconsin, 2019

Characteristic	n/N (%)			
	THC-containing products only (N = 25)	Nicotine-containing products only (N = 11)	Both THC- and nicotine-containing products (N = 50)	Total (N = 86)
**Age group (yrs)**				
<18	5/25 (20)	3/11 (27)	11/50 (22)	**19/86 (22)**
18–24	7/25 (28)	4/11 (36)	27/50 (54)	**38/86 (44)**
25–34	7/25 (28)	3/11 (27)	9/50 (18)	**19/86 (22)**
≥35	6/25 (24)	1/11 (9)	3/50 (6)	**10/86 (12)**
**Gender**				
Male	22/25 (88)	8/11 (73)	38/50 (76)	**68/86 (79)**
Female	3/25 (12)	3/11 (27)	12/50 (24)	**18/86 (21)**
Race/Ethnicity*				
White, non-Hispanic^†^	13/22 (59)	8/11 (73)	39/46 (85)	**60/79 (76)**
Black, non-Hispanic^†^	2/22 (9)	2/11 (18)	3/46 (7)	**7/79 (9)**
Other, non-Hispanic^†^	0/22 (0)	0/11 (0)	2/46 (4)	**2/79 (3)**
Hispanic^†^	7/22 (32)	1/11 (9)	2/46 (4)	**10/79 (13)**
**Other characteristics**				
Admitted to ICU^§^	12/19 (63)	5/8 (63)	25/44 (57)	**42/71 (59)**
Smoked combustible marijuana^¶^	12/24 (50)	5/11 (45)	26/48 (54)	**43/83 (52)**
Smoked combustible tobacco^¶^	3/24 (13)	4/11 (36)	13/48 (27)	**20/83 (24)**

**Abbreviations:** ICU = intensive care unit; THC = tetrahydrocannabinol.

* Information missing for seven patients.

^†^ Blacks, whites, and persons of other races were non-Hispanic; Hispanic persons could be of any race.

^§^ Information missing for 15 patients.

^¶^ Information missing for three patients.

Among the 86 interviewed patients, 75 (87%) reported using e-cigarette products containing THC, the principal psychoactive component of cannabis, during the 3 months preceding illness; 61 (71%) reported using nicotine-containing products; 50 (58%) reported using both THC- and nicotine-containing products. Twenty-five (29%) patients reported exclusive use of THC-containing products, whereas 11 (13%) reported exclusive use of nicotine-containing products (Table 2). Demographic characteristics of patients were similar among those who reported exclusive use of THC-containing products, exclusive use of nicotine-containing products, or use of both types of products (Table 1).

**TABLE 2:** Electronic cigarette (e-cigarette), or vaping, product use behaviors in the 3 months prior to illness onset in patients with lung injury associated with e-cigarette use — Illinois and Wisconsin, 2019

Product use and behaviors	No. (%)		
	Illinois (n = 48)	Wisconsin (n = 38)	Total (N = 86)
**THC-containing product use**			
Any use	39 (81)	36 (95)	**75 (87)**
Exclusive use	13 (27)	12 (32)	**25 (29)**
Dank Vapes use	33 (73)	24 (63)	**57 (66)**
**Nicotine-containing product use**			
Any use	35 (73)	26 (68)	**61 (71)**
Exclusive use	9 (19)	2 (5)	**11 (13)**
**Both THC- and nicotine-containing product use**	26 (54)	24 (63)	**50 (58)**
At least daily use of e-cigarette products*			
THC-containing products	29 (60)	20 (53)	**49 (57)**
Nicotine-containing products	27 (56)	18 (47)	**45 (52)**
**Devices used with e-cigarette products^†^**			
Device designed for prefilled cartridge use	43 (91)	35 (92)	**78 (92)**
Tank designed to be filled with product	7 (15)	11 (29)	**18 (21)**
Dab rig or a dab pen	7 (15)	7 (18)	**14 (16)**
**No. of e-cigarette product brands reported per product type user^†^**			
THC brands per THC user,^§^ mean (range)	2.1 (1–7)	2.1 (1–7)	**2.1 (1–7)**
Nicotine brands per nicotine user,^¶^ mean (range)	1.3 (1–3)	1.3 (1–4)	**1.3 (1–4)**
**Packaging of e-cigarette products used**			
No./total of THC products (%) that were packaged, prefilled cartridges	69/72 (96)	80/83 (96)	**149/155 (96)**
No./total of nicotine products (%) that were packaged, prefilled cartridges	32/35 (91)	29/44 (66)	**61/79 (77)**

**Abbreviation:** THC = tetrahydrocannabinol.

* The denominator used here is all patients, not just those who reported using THC- or nicotine-containing products.

**^†^** Patients could report using more than one type of device or product, thus the percentage totals sum to >100%.

^§^ Patients were counted as THC users if they reported use of at least one THC-containing e-cigarette product in the past 3 months.

^¶^ Patients were counted as nicotine users if they reported use of at least one nicotine-containing e-cigarette product in the past 3 months.

The chemical contents of reported THC-containing products are unknown. However, urinary THC screens were obtained for 32 patients who reported using THC-containing products, 29 (91%) of which were positive for THC; two patients who did not report using THC-containing e-cigarette products, out of four tested, also had positive urinary THC screens; one of these patients reported smoking combustible marijuana. Urinary THC levels for four patients who reported using THC-containing products exceeded 400 ng/ml, indicating intensive use of THC or THC-containing products (*4**,**5*). In Wisconsin, eight patients initially denied using THC-containing products in interviews, but five (63%) were later found to have used THC through review of medical charts, reinterview, or cross-referencing with friends who were also interviewed as patients.

Among the 86 interviewed patients, 234 unique e-cigarette, or vaping, products labeled with 87 different brand names were reported. Nicotine-containing product users reported a mean of 1.3 different nicotine brands (range = 1–4), and THC-containing product users reported a mean of 2.1 different THC brands (range = 1–7). Among 155 THC-containing products reported, nearly all (149, 96%) were packaged, prefilled cartridges, whereas 61 (77%) of 79 nicotine-containing products were sold as prefilled cartridges or “pods.” No patients reported adding other ingredients to the e-cigarette products they used. Although no single brand name was reported by all patients, a prefilled THC cartridge sold under the brand name Dank Vapes was reported by 57 (66%) patients (Figure). In Wisconsin, two groups of friends (two patients in one group and three in the second group) who became ill after using THC-containing cartridges specifically reported sharing Dank Vapes cartridges. Dank Vapes was the only e-cigarette product reported by one of the patients.

**FIGURE:**
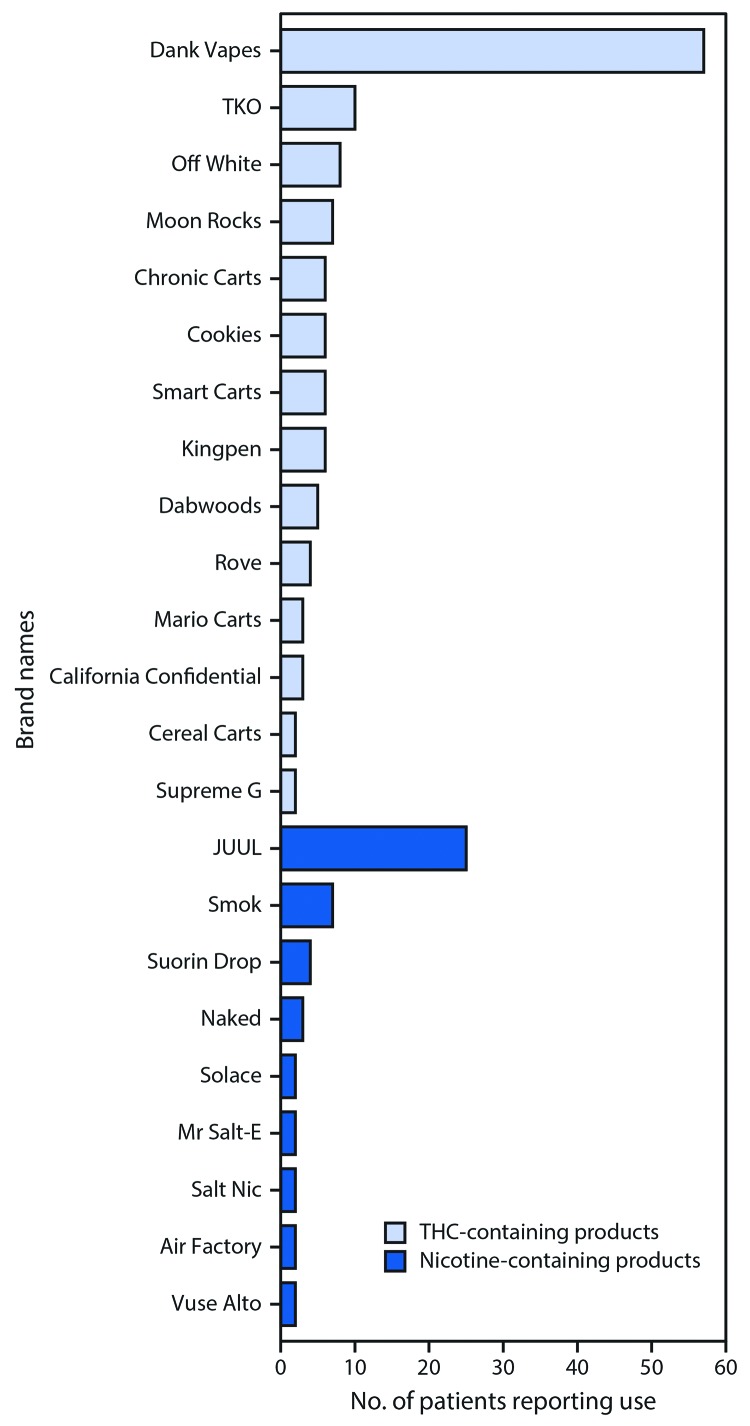
Frequently reported brand names of tetrahydrocannabinol (THC)- and nicotine-containing electronic cigarette (e-cigarette), or vaping, products*^,†,§^ reported by patients with lung injury^¶^ — Illinois and Wisconsin, 2019 * Two brands of cannabidiol are not shown (each brand reported by one patient). ^†^ 30 other THC-containing brands (including three brands of THC wax for “dabbing”) were only reported by one patient each. ^§^ 22 other nicotine-containing brands were only reported by one patient each. ^¶^ Data are presented from interviews conducted with 86 of 127 patients with lung injury associated with e-cigarette use, or vaping.

Among 112 THC-containing products for which the source was reported, 100 (89%) were acquired from informal sources (e.g., friends, family, school, dealers, or off the street). The remaining 12 were bought at an out-of-state cannabis dispensary (six), online (five), or from a vape or tobacco shop (one). In contrast, among 81 nicotine-containing products, 40 (49%) were obtained from a vape or tobacco shop, 22 (27%) from gas stations or convenience stores, 14 (17%) from friends or family, and five (6%) online.

A variety of e-cigarette and vaping device types (*6*) were used by patients to aerosolize THC- or nicotine-containing products. Overall, 78 (92%) of 85 patients reported using a device designed to aerosolize prefilled cartridges or pods. Within this category of vaping devices, some were closed-pod systems (also known as “mods”) designed for use with proprietary nicotine-containing products (e.g., JUUL); however, most were universal “vape pens” that are adaptable to the prefilled THC cartridges reported by many patients. Use of devices with a tank designed to be filled with nicotine-containing liquid or THC oil was reported by 18 (21%) patients, and 14 (16%) reported aerosolizing THC concentrates, known as waxes or “dabs,” using either a “dab rig” or a “dab pen” device.^†^

Patients reported frequent daily use of e-cigarette products; among 75 users of THC-containing products, 49 (65%) reported using these products at least daily, and 45 (74%) of 61 nicotine-containing product users reported at least daily use of these products. Where more detailed information on frequency of use was provided, 21 (41%) of 51 THC-containing product users and 30 (65%) of 46 nicotine-containing product users reported use of at least one such product five or more times a day. In addition to e-cigarette products, among 83 patients who provided information on combustible product use, 43 (52%) reported smoking combustible marijuana, and 20 (24%) reported smoking combustible tobacco.

Only four (5%) of 86 interviewed patients reported prescription drug misuse or illicit drug use other than THC. Two patients reported using LSD, one reported misusing dextroamphetamine-amphetamine (Adderall), and one reported misusing oxycodone. Urinary toxicology screens were positive for substances other than THC (and for other substances that could not be explained by the medical treatment these patients had received) in six of 31 patients, including two patients who tested positive for benzodiazepines and opioids, one for benzodiazepines alone, one for opioids alone, one for amphetamines, and one for unspecified narcotics.

## Discussion

In this series of in-depth interviews with 86 e-cigarette– or vaping-associated lung injury patients in Illinois and Wisconsin during July–September 2019, patients reported a wide range of e-cigarette products; however, the vast majority reported using illicit THC-containing products sold as prefilled cartridges and obtained from informal sources. Although no single brand or product was definitively identified, a high percentage of patients reported using Dank Vapes cartridges. Dank Vapes appears to be the most prominent in a class of largely counterfeit brands, with common packaging that is easily available online and that is used by distributors to market THC-containing cartridges with no obvious centralized production or distribution (*7*).

Previous reports highlighted that patients with lung injury associated with e-cigarette use have used both THC- and nicotine-containing products (*1**,**3**,**8**,**9*). The additional information presented here regarding the range and diversity of brands used by patients, acquisition patterns, and frequency of use helps to formulate hypotheses about the possible etiology of this outbreak. In particular, the high level of use of prefilled THC cartridges, used in a range of different devices, suggests that the cartridges might play an important role.

The findings in this report are subject to at least four limitations. First, interviews were not available for one third of patients; this nonresponse rate might introduce selection bias, although the demographics of the 86 interviewed patients were similar to those of all 127 patients. Second, because information was self-reported, there is the possibility that social desirability bias might affect reporting, particularly of illicit products; nonmedical THC use is currently illegal in both Illinois and Wisconsin. In this analysis, some patients did not disclose THC-containing product use to clinicians until late in their hospital admission or until a urinary THC screen was performed. Third, the time between urinary toxicology testing and last reported use of an e-cigarette product was not consistent and might explain the three negative results in patients who reported using THC-containing products. Finally, these data are largely drawn from patients living in the northeastern region of Illinois and southeastern region of Wisconsin, and therefore might not be generalizable to other states; however, the age and gender distribution of is consistent with nationwide trends (*2**,**3*).

The findings document that many, but not all, patients with lung injury associated with use of an e-cigarette product reported using THC-containing products. Similar findings have been noted in the national data, which include some of the data presented here (*2*). These data also reveal a predominant use of prefilled THC cartridges sold through informal and unregulated markets, although the origin of these products further back in the production and distribution chain is unknown. In addition, these data do not elucidate whether the causative exposure is THC itself or a substance associated with prefilled THC cartridges, such as a cutting agent or adulterant. Ascertaining the importance of these products in contributing to the current outbreak will require data from multiple states and analysis at the national level.

Given the number and diversity of products reported overall and by individual patients, as well as the high frequency of patients using both THC- and nicotine-containing products, the epidemiologic investigation could benefit from additional information, including product testing and traceback of e-cigarette products to identify the ultimate source of the outbreak. The Illinois Department of Public Health and the Wisconsin Department of Health Services are collaborating with CDC on a large nationwide public health response and with the Food and Drug Administration to coordinate laboratory testing of products associated with this outbreak. While this investigation is ongoing, CDC recommends that persons consider refraining from using e-cigarette, or vaping, products, particularly those containing THC.

**Summary**

**What is already known about this topic?**

An outbreak of lung injury of unknown source associated with electronic cigarette (e-cigarette) use is ongoing in the United States.

**What is added by this report?**

Interviews about e-cigarette use were completed with 86 patients in Illinois and Wisconsin. Use of tetrahydrocannabinol (THC)-containing e-cigarette products, the majority of which were prefilled cartridges obtained from informal sources, was reported by 87% of patients during the 3 months preceding illness.

**What are the implications for public health practice?**

The cause of this outbreak is unknown but might be related to prefilled THC cartridges. While this investigation is ongoing, CDC recommends that persons consider refraining from using e-cigarette, or vaping, products, particularly those containing THC. Additional information from product testing and traceback could help to determine the source of the outbreak and prevent future illnesses.

## Footnotes

* These authors contributed equally.

† Dabbing is a process that allows the user to inhale a high concentration of THC by vaporizing extracts of a concentrate that has been placed on a hot surface.
